# Family impact and economic burden among caregivers of children with chronic kidney disease in Assiut, Egypt

**DOI:** 10.1186/s42506-020-00058-7

**Published:** 2020-10-07

**Authors:** Manal M Darwish, Shimaa Hosny Hassan, Samaher Fathy Taha, Hosnia Said Abd El-Megeed, Taghreed Abdul-Aziz Mohammad Ismail

**Affiliations:** 1 Public Health and Community Medicine Department, Faculty of Medicine, Assiut University, Assiut, Egypt; 2 Assiut Children Hospital, Faculty of Medicine, Assiut University, Assiut, Egypt

**Keywords:** Pediatric, Chronic renal disease, Financial impact, Caregivers

## Abstract

**Background:**

Chronic disease greatly increases children’s dependency on parents/caregivers (usually mothers) as they face new problems associated with caring for a child with chronic disease. Thus, chronic kidney disease (CKD) presents a burden for children and their families that last throughout life in different aspects. This study aimed to assess family impact and economic burden of chronic kidney disease (CKD) in children on their families.

**Methods:**

A cross-sectional study was carried out on 250 caregivers of children with CKD attending tertiary care hospital/health insurance clinics in Assiut, Egypt using PedsQL™ family impact module (FIM) for assessing family impact and economic burden between January and May 2018.

**Results:**

Seventy six percent of caregivers shared paying for treatment with health insurance while 14% paid the total expenses out of their pockets. Although the majority (87.2%) of caregivers suffered different degrees of financial hardship, more than 60% of them had no coping strategy. The regression module showed that responding caregiver, degree of financial hardship, treatment modality, and socioeconomic class were significant predictors of total FIM (*β* = 0.38, *P* < 0.001; *β* = 0.28, *P* < 0.001; *β* = 0.22, *P* < 0.001; *β* = 0.13, *P* = 0.006 respectively).

**Conclusion:**

Most caregivers were involved in paying for treatment of their children either totally or sharing with health insurance and suffered different degrees of financial hardship. Mothers, caregivers with great financial hardship, and caregivers of children on dialysis had the lowest scores of PedsQL™ FIM. There is a high need for expansion of health insurance umbrella to reduce financial hardship together with continued multidimensional support to families.

## Introduction

Childhood chronic diseases usually affect family functioning. Chronic disease greatly increases children’s dependency on parents/caregivers (usually mothers) as they face new problems associated with caring for a child with chronic disease [1]. Furthermore, family plays an important role in the child’s adaptation and coping with his/her chronic disease. Therefore, children’s health-related quality of life (HRQoL) assessments need to be supplemented with family functioning assessments [2].

The assessment of family impact of chronic childhood illness is extremely useful to identify the need for family education, psychological intervention, and social support. This assessment is also important for health care professionals and policy makers to improve the HRQoL of children and their caregivers [3].

One of the most chronic diseases which can affect the quality of life (QoL) of children and their families is chronic kidney disease (CKD) [4]. Caring for those children places significant economic stress on caregivers: health care costs, disruption to work, travel expenses, and out-of- pocket payments. Financial stress experienced by caregivers contributes to their ability to provide care for their children and affect parent’s QoL [5].

Most of the previous studies evaluated the impact and quality of life for caregivers of children with other chronic diseases such as type 1 diabetes [6], chronic gastrointestinal disorders [2], chronic pain [7], sickle cell disease [8], disabilities [9], asthma, and heart disease [3]. Studies that correlate between the degree of financial hardship and QoL of caregivers of children with CKD in Egypt could not be traced after careful searching.

After careful searching, no studies that investigated the correlation of the degree of financial hardship and QoL of caregivers of children with CKD in Egypt were traced. This study aims to determine the family impact of pediatric CKD (physical, psychological, and social) and to assess the economic burden of children with CKD on their families.

## Methods

### Study design, setting, and population

A cross-sectional study was conducted at two sites: health insurance clinic and Assiut University Children Hospital (nephrology outpatient clinic, nephrology department, and dialysis unit) in Assiut city, Upper Egypt, between January and May 2018.

The university hospital with its all levels staff carders (about 65 personnel) provides care for 450 inpatients (36 with ESRD receiving dialysis and 414 on conservative treatment) and an approximately 20 outpatients/day suffering from both acute and chronic kidney problems (chronic is our target). All the recommended battery of services—except kidney transplantation which is offered at Abu El-Reesh children Hospital (Cairo University)—is provided and payment for the services is via health insurance, government expenses, out of pocket, or mixed, whereas health insurance clinic with less staffing (3 personnel) works only 2 days a week and provides only conservative treatment (dialysis cases are referred to the university hospital) to approximately 25–30 patients/week. All medical treatments provided in this clinic is paid via health insurance.

### Inclusion criteria

The inclusion criteria are as follows: any caregiver of child (8 to ≤ 18 years) diagnosed with CKD who agreed to participate in the study and receive any management modality (renal replacement therapy, conservative treatment, dialysis or kidney transplantation).

### Sample size and sampling technique

The sample size was calculated using OpenEpi, Version 3, open source calculator—SSPropor with a population size of 1000 and expected frequency of financial hardship in affected families of 28% [10]; the calculated sample size was 237 at 95% confidence level. The researchers increased the number to 250 to safeguard against incomplete and missing data.

- The sample included all end-stage renal disease (ESRD) cases (36 cases) registered in the dialysis unit in the Children University Hospital which is the only unit for all children with ESRD requiring dialysis.
- For children on conservative treatment (214 cases), a systematic random sample from eligible attendants was selected and a proportional allocation based on the days of work of the 2 clinics resulted in 169 cases from Assiut University Children Hospital and 45 cases from the health insurance clinic.

### Data collection and tools

Data were collected through personal interviews with caregivers of eligible children (mainly the mothers). Four tools were used in the study.

*Tool I*: A questionnaire to collect demographic and clinical data which include name, age, sex, residence, educational level, work status of mother, socioeconomic class, and data about CKD (family history, duration, stage, and treatment modality).

*Tool II*: *Family Socio-economic Scale*, *revised version 2010* [11]. It assesses the socioeconomic status of the family and consists of 4 dimensions, parents’ level of education (8 items), parents’ occupation (2 items), total family monthly income (6 items), and lifestyle of the family (12 items). The item of income was modified according to the rate of inflation [12] (details in Additional file 1).

*Tool III*: *Assessment of the impact of pediatric CKD on families using the Arabic version of the PedsQL™ family impact module (FIM) version 2* which is composed of 36 items in 8 dimensions including physical function (6 items), emotional function (5 items), social function (4 items), cognitive function (5 items), communication (3 items), worry (5 items), daily activities (3 items), and family relationships (5 items). It is a 5-point rating Likert scale ranging from “never” to “almost always” as follows: 0 if it is never a problem and 4 if it is almost always a problem. Items were then reverse-scored and linearly transformed to a 0–100 scale (0 = 100, 1 = 75, 2 = 50, 3 = 25, 4 = 0) so that higher scores indicate better functioning (less negative impact) [13] (details in Additional file 2). The FIM yields 3 “mean” summary scores = sum of the items over the number of items answered. The mean of total score is the sum of all 36 items divided by the number of items answered. The Parent HRQoL Summary Score (20 items) in the physical, emotional, social, and cognitive functioning scales. The Family Functioning Summary Score (8 items) is computed as the sum of the items divided by the number of items answered in the daily activities and family relationships scales [14] (details in Additional file 3). The PedsQL™ FIM demonstrated accepted reliability in all summary and scale scores as evidenced by Cronbach’s alpha coefficients greater than 0.8 [8]. Overall, the FIM was found to demonstrate excellent internal consistency and criterion validity within a community sample in both total and summary scales [15].

The PedsQL™ FIM (Arabic version) showed excellent internal consistency reliability for the total PedsQL™ Family Impact Scale as well as its subscales. Cronbach’s alpha score was above 0.93 for the total PedsQL™ Family Impact Scale [16].

*Tool IV*: *Assessment of economic burden on family using a questionnaire including the following items*: financial hardship, work disruptions, search for an additional job, coping strategies, travel expenses, and type of payment for treatment [10–18].

### Operational definitions

- Socioeconomic status (SES) refers to the level of the social and economic position of an individual within the society as reflected by various indicators. These include income, education, occupation, place of residence, access to basic services, and the availability of infrastructure, among others [19].
- Severity (staging) of CKD: Children with CKD were classified according to the glomerular filtration rate (eGFR) using the Updated Bedside Schwartz formula as follows:

eGFR (ml/min per 1.73 m^2^) = 41.3 × (height/Scr)

where height is in meters and Scr (serum creatinine) is in mg/dl [20].

- According to the eGFR, children with CKD were classified as follows [21]:

**Table:** 

**Stage**	**GFR (ml/min/1.73m**^**2**^**)**	**Terms**
Stage 1	> 90	Normal or high
Stage 2	60–89	Mildly decreased
Stage 3a	45–59	Mildly to moderately decreased
Stage 3b	30–44	Moderately to severed decreased
Stage 4	15–29	Severely decreased
Stage 5	< 15	Kidney failure

### Ethical considerations

The study protocol was approved by the ethical review board of the Faculty of Medicine, Assiut University (Reference # IRB00008718). The aim of the study was explained to caregivers before starting the data collection. Voluntary participation of caregivers of the child was assured. Verbal informed consent was obtained from both caregivers and children before participating in the study (verbal consenting process was accepted by the ethical review board of the Faculty of Medicine, Assiut University, due to the high prevalence of low education levels, especially in rural areas, the prevailing culture of fear of signing any document, and the nature of research, which is not interventional, only questionnaire-based). Privacy and confidentiality of all data was assured.

### Data management and statistical analysis

Data entry, cleaning, analysis, and recoding (if needed) was done using the Statistical Package for Social Science (SPSS Inc., Chicago, IL, USA) version 20 [22]. Descriptive statistics were calculated as the mean and SD for continuous variables and as frequency and percentages for categorical variables. Chi-squared (*χ*^2^) and Fisher’s exact tests were used as the tests of significance for categorical variables. One-way ANOVA test was used for the three or more group comparison of continuous variables and the Student *t* test for the two groups. Multivariate linear regression analysis was used to identify the different predictors of family-functioning scores. The odds ratio was calculated as a measure of association at 95% confidence limit, and statistical significance level was considered when *P* value ≤ 0.05 for all statistical tests.

## Results

Table 1 shows the sociodemographic and clinical characteristics of the sample children with CKD. The mean age of the children with CKD was 11.9 ± 3.1 years. Males represented approximately two thirds of the study sample (67.2%). The duration of CKD exceeded 5 years in 36% and most of children in the study (85.6%) received conservative treatment while 14.4% were on regular hemodialysis. Regarding CKD severity, about half of the studied children (50.2%) were in stage 5 and 26.6% were in stage one of CKD.

**Table 1:** Sociodemographic and clinical characteristics of children with CKD, Assiut, Upper Egypt, 2018

Variables	Number (250)	Percentage (%)
**Age**		
Mean ± SD (range)	11.9 ± 3.1 (8–18)	
**Sex**		
Male	168	67.2
Female	82	32.8
**Residence**		
Urban	38	15.2
Rural	212	84.8
**Education level**		
No schooling	10	4.0
Primary	144	57.6
Preparatory	65	26
Secondary	31	12.4
**Mother’s working status**		
Housewife	236	94.4
Working mother	14	5.6
**Socioeconomic level**		
Low	42	16.8
Middle	164	65.6
High	44	17.6
**Duration of CKD**		
≤ 5 years	158	63.2
> 5 years	92	36.8
**Treatment modality**		
Conservative	214	85.6
Dialysis	36	14.4
**Stage of CKD**^a^	*N* = (222)	
Stage 1	59	26.6
Stage 2	20	9
Stage 3	15	6.8
Stage 4	16	7.2
Stage 5	112	50.4

^a^Stage of CKD: total = 222 (28 children had no available kidney function tests)

Table 2 shows that most caregivers (76%) paid for treatment through both health insurance and out-of-pocket, 14% were obliged to pay for treatment from their out-of-pocket, 6.8% paid totally by health insurance, and only 3.2% of children were treated on the government expense via ministerial decrees. Most caregivers of the studied children with CKD (60%) reported having great financial hardship because of CKD of their children, and only 12.8% had no financial hardship. The main coping strategies used by caregivers were sold property (21.1%) followed by taking out a loan (11.5%).

**Table 2:** Economic impact of CKD on families of affected children, Assiut, Upper Egypt, 2018

Characteristics	Number (250)	Percentage (%)
**Type of payment for treatment**		
Health insurance	17	6.8
Out-of-pocket	35	14
Both health insurance and out-of-pocket	190	76
Treatment on government expense	8	3.2
**Work disruptions**		
Yes	20	8
No	230	92
**Need for additional job by a family member**		
Yes	35	14
No	215	86
**Need to travel to another governorate**		
Yes	98	39.2
No	152	60.8
**Accommodation expenses**		
Yes	70	28
No	180	72
**Degree of financial hardship**		
No	32	12.8
Little	19	7.6
Moderate	49	19.6
Great	150	60
**Coping strategies**	*N* = (218)^a^	
None	137	62.8
Sold property	46	21.1
Took out a loan	25	11.5
Forego making a big purchase	6	2.8
Fundraising	4	1.8

^a^The total number was 218 because 32 caregivers had no hardship

Table 3 shows that the mean score of the total PedsQL™ FIM was 51.9 ± 22.2, the parent HRQoL summary score was 48.7 ± 25.2, and the family summary score was 72.7 ± 27.01. The worry score was the least, while the highest was the family relationships.

**Table 3:** PedsQL™ Family Impact Score in caregivers of children, with CKD Assiut, Upper Egypt, 2018

Scale	Mean ± SD
Physical	51.1 ± 32.5
Emotional	42.1 ± 26.2
Social	61.2 ± 36.2
Cognitive	42.8 ± 31.5
Communication	52.3 ± 32.5
Worry	24.4 ± 21.3
Daily activities	69.1 ± 34.2
Family relationships	74.5 ± 30.6
**Parent HRQoL summary score**	**48.8 ± 25.2**
**Family summary score**	**72.7 ± 27**
**Total score**	**51.9 ± 22.2**

Table 4 shows that there were statistically significant differences in parental HRQoL, family summary, and total FIM by responding caregiver, degree of financial hardship, and treatment modality where mothers, those with high financial hardship, and children on dialysis had the lowest scores. Also, those with low SES had significantly lower scores in both parental HRQoL and total FIM while disease duration and CKD stage had no significant effects. Caregivers of children with stage 5 of CKD reported the lowest scores compared with caregivers of children in other stages of CKD.

**Table 4:** Mean scores of PedsQL™ Family Impact Module in caregivers of CKD children, Assiut, Upper Egypt, 2018

	Parental HRQoL Mean ± SD	Family summary Mean ± SD	Total FIM Mean ± SD
**Caregiver**			
Mother	37.4 ± 22.6	63.9 ± 28.8	41.9 ± 20.3
Father	61.4 ± 19.3	86.4 ± 15.2	62.9 ± 15.7
Brother	59.9 ± 26.4	76.5 ± 27.6	62.5 ± 24.4
Sister	47.4 ± 29.1	55.8 ± 28.5	47.9 ± 24.8
***F*** **value**^**a**^	17.5	12.5	19
***P*** **value**	< 0.001*	< 0.001*	< 0.001*
**Degree of financial hardship**			
No	66.2 ± 18.6	82.5 ± 24.1	66.9 ± 17.9
Little	64.1 ± 23.7	86.7 ± 19.9	59.6 ± 17.2
Moderate	58.3 ± 20.7	58.3 ± 20.7	59.6 ± 17.2
High	39.9 ± 24	66.5 ± 28.7	44.5 ± 21.3
***F*** **value**^**a**^	29.5	10.9	27.2
***P*** **value**	< 0.001*	< 0.001*	< 0.001*
**Treatment modality**			
Conservative	52.4 ± 24.1	78.1 ± 22	55.4 ± 20.6
Hemodialysis	27.2 ± 20.4	40.6 ± 31.8	31.8 ± 20.8
***T*** **value**^b^	5.9	8.8	6.4
***P*** **value**	< 0.001*	< 0.001*	< 0.001*
**Socioeconomic level**			
Low	40.5 ± 25.6	63.4 ± 29.6	43.8 ± 23.1
Middle	48.9 ± 24.9	48.9 ± 24.9	52.8 ± 21.6
High	55.6 ± 23.8	77.7 ± 24.6	56.8 ± 21.9
***F*** **value**^**a**^	3.9	2.6	4.1
***P*** **value**	0.02*	0.07	0.01*
**Duration of CKD**			
≤ 5 years	46.4 ± 25.1	71 ± 28.6	71 ± 28.6
> 5 years	52.6 ± 25	75.5 ± 23.9	55.3 ± 21.9
***t*** **value**^b^	1.9	1.3	1.8
***P*** **value**	0.06	0.2	0.07
**Stage of CKD**			
Stage 1	47.2 ± 27.2	77.1 ± 20.9	52.4 ± 22.4
Stage 2	61.5 ± 21.9	75.9 ± 31.6	62.3 ± 22
Stage 3	46.5 ± 25.7	80.8 ± 17.6	49.3 ± 23.6
Stage 4	51.3 ± 20.6	84.9 ± 13.2	54.9 ± 16.1
Stage 5	45.6 ± 24.8	65.5 ± 31.2	48.5 ± 22.1
***F*** **value**^**a**^	1.9	2.4	1.89
***P*** **value**	0.12	0.052	0.11

^a^ANOVA test (post hoc) was used

^b^Independent sample *t* test was used

*Significant

Table 5 shows the linear regression module for the predictors of the total PedsQL™ FIM score. The variables of: responding caregiver (*P* < 0.001, CI = 4–7.1), degree of financial hardship (*P* < 0.001, CI = 3.5–8.1), treatment modality (*P* < 0.001, CI = 8.6–22.4), and socioeconomic class (*P* = 0.001, CI = 2.8–10.4) were significantly associated with the total PedsQL™ FIM score, while there was no significant difference with the stage of CKD. About 42% of the variations in the total impact score were explained by these explanatory variables (*R*^2^ = 0.420): fathers and brothers (as caregivers of the sick child), caregivers suffering no or little financial hardship, caregivers of children on conservative treatment, and those from high socioeconomic class got higher PedsQL™ FIM scores.

**Table 5:** Predictors of total PedsQL™ Family Impact Module score in parents of CKD children, Assiut, Upper Egypt, 2018

Variable	Beta	***t***	Sig.	95% CI
Responding caregiver	0.38	7	**< 0.001**	4–7.1
Degree of financial hardship	0.27	4.9	**< 0.001**	3.5–8.1
Treatment modality	0.26	4.4	**< 0.001**	8.6–22.4
Socioeconomic level	0.18	3.4	**0.001**	2.8–10.4
Stage of CKD	0.04	0.6	0.51	0.9–1.9
**Constant**		6.2	**< 0.001**	21.5–41.4
***R*** **square**^**a**^	0.420			

^a^Simple linear regression analysis

## Discussion

Because most patients with CKD especially children need comprehensive support and care, their caregivers feel more physical, psychological, and financial stress. Unfortunately, few studies have been conducted to measure the QoL of caregivers of children with CKD [23].

Our results showed that work disruption occurred in only 8% of caregivers, as the primary caregiver is usually the mother and 94% of mothers in our study were housewives. Also, only 14% of caregivers sought an additional job, which can be attributed to unavailability of jobs, especially part-time jobs. Furthermore, most caregivers in our study were illiterate and of lower educational levels, which make their chances of finding jobs low. Two previous studies show that caregivers of children with ESRD were unemployed and financially supported by another member of the family [24, 25].

In our study, more than one third of caregivers reported need to travel to another governorate to receive medical care for their children and some of them were obliged to pay for long time accommodation. In most cases, travel was primarily for kidney biopsy, consulting another nephrologist or for renal transplantation. This was in agreement with caregivers of children with cancer who reported need for extra payment for travel, lodging, and meals away from home as reported by Miedema et al. [18].

Regarding the method of payment, a small percentage (6.8%) of caregivers were completely covered by health insurance and only 3.2% of children were treated on government expense by a ministerial decree while about three quarters (76%) shared paying treatment through both health insurance and out-of-pocket. The remaining 14% had to pay for treatment out-of-pocket. This is because health insurance covers the cost of some drugs and investigations; the family must cover the rest of medical treatment from out-of-pocket. The total FIM and parental HRQoL scores were more severely impaired than family functioning.

Lower scores in most of the scales in this study reflect the inability of caregivers to deal with the different stressors due to caring for their diseased children and also the limited facilities and services available for diseased children and their caregivers. In addition, this may relate directly to the fact that most of the burden is falling on mothers (50.4%).

The total PedsQL™ FIM and parental HRQoL of caregivers of children with CKD was lower in our population compared with other studies of caregivers of children with other chronic diseases (asthma [26], osteogenesis imperfecta [27], sickle cell disease [8], asthma and heart diseases [3], chronic gastrointestinal disorders [2], chronic pain [7], nephrotic syndrome [28], disabilities [29], and different chronic conditions [15]).

Notably, the lowest score in our study was the worry function. This may be due to the nature of CKD and its anticipated progression to ESRD and related premature mortality, in addition to worry about the efficacy, side effects of treatment, and comorbidities associated with CKD. This was consistent with results of studies in caregivers of children with nephrotic syndrome, one of the major causes of CKD in our children, who also reported the lowest score in worry function [28, 30].

On the other hand, the highest score in our study was in the family relationships (74.5 ± 30.6). This may be due to the way the Egyptians react in coping with chronic disease, especially in children, as family cooperation and cohesion is a common feature in facing difficulties. However, it was not possible to establish whether these positive family relationships were found before the diagnosis of CKD or not.

Compared to fathers and brothers, mothers and sisters of children with CKD reported significantly lower scores in total FIM, parental HRQoL, and family summary scores. This is because mothers usually carry a disproportionate number of parenting demands (such as monitoring the child’s compliance to treatment, follow-up visits, hospitalization, diet, and fluid requirements) and lifestyle disruptions (such as absence from work or quitting job). In addition, mothers are responsible for the daily care of other family members along with their ill child’s health and functioning. Our findings were similar to those reported in other populations where mothers of children with chronic pain, cancer, and disabilities reported significantly worse total FIM, parent HRQoL, and family summary scores than fathers [7, 29, 31]. Our findings are also supported by another research using the Ulm Quality of Life Inventory for parents, in which mothers of CKD children achieved significantly lower total, physical, emotional, and daily functioning than fathers [32].

There were statistically significant differences in the PedsQL™ FIM scores in all domains of QoL by the degree of financial hardship where caregivers who suffer from great financial hardship due to their children CKD acquired the least score in all domains of FIM. As a result of poverty and working mainly in low-paying jobs (67.6% of fathers work in simple and non-technical professions while 94% of mothers were not working for cash), caregivers of children with CKD in our study tend to achieve lower QoL scores with increase in financial hardship.

Regarding treatment modality, our study revealed that there were statistically significant differences between conservative and dialysis groups in all domains of PedsQL™ FIM, except for communication and worry domains. In all domains, caregivers of children who received conservative treatment achieved higher scores than caregivers of children on hemodialysis. This can be explained by the burden of hemodialysis sessions (three times weekly and each session lasts about 3–4 h), transportation from remote areas, restriction of activities, work disruption, cost of dialysis and other medications plus psychological upset and worry about complications of the invasive dialysis-related procedures. A study in Germany using the Ulm Quality of Life Inventory for Parents (ULQIE) showed that parents of children undergoing dialysis also reported the lowest scores on all scales, whereas parents of children undergoing conservative treatment reached the higher HRQoL scores [32].

Though not reaching the level of significance, caregivers of children with CKD duration > 5 years reported higher scores in parental HRQoL family summary than those of children with CKD duration ≤ 5 years. This phenomenon is called “response shift” which means that with passage of time caregivers learned how to cope with and adjust to their child illness and thus report better QoL [33]. In contrast to our results, a study of family impact of children with ESRD revealed higher scores of the PedsQL™ FIM with short duration (< 5 years) than longer duration (≥ 5 years) [23]. This result suggests that a longer duration of ESRD can impose additional burden on parents or caregivers especially when their children reach end-stage of the disease.

Similarly, there were statistically significant differences in the total PedsQL™ FIM and parent HRQoL scores by social class where caregivers from the higher socioeconomic level reported higher scores in all domains than those in the middle and low socioeconomic level. Thus, with improvement in socioeconomic conditions, caregivers tend to adjust better to their children CKD and so achieve higher QoL scores.

### Strengths and limitation of the study

To our knowledge, this study is the first to use PedsQL™ Family Impact Module to study the family impact of CKD in children in all stages of CKD not only ESRD [23]. However, this study has some limitations: caregivers were recruited in the study during a routine follow-up visit or after hemodialysis sessions and therefore the reported scores are not in direct response to disease exacerbation. Research will be required to assess the short-term impact of such events on caregivers. Another limitation is the absence of a control group due to cross sectional nature of the study. However, the QoL scores of parents of children with CKD were lower than caregivers of healthy children.

## Conclusion

Most caregivers were involved in paying for treatment of their children either totally or sharing with the health insurance and suffered different degrees of financial hardship. Significant predictors of the Total FIM were the responding caregiver, degree of financial hardship, treatment modality, and socioeconomic class.

The expansion of the umbrella of health insurance is an essential need to reduce the out of pocket payment with multidimensional support for families of children with CKD in different aspects.

## Supplementary information

**Additional file 1.**

**Additional file 2.**

**Additional file 3.**

## Data Availability

Datasets used in the current study are available from the corresponding author on reasonable request.

## Abbreviations

CKD: Chronic kidney disease
ESRD: End-stage renal disease
FIM: Family impact module
eGFR: Glomerular filtration rate
HRQoL: Health-related quality of life
PedsQL™ FIM: Pediatric quality of life™ family impact module
QoL: Quality of life
SPSS: Statistical Package for Social Science
ULQIE: Ulm Quality of Life Inventory for Parents
