# Supplementary material for: Family impact and economic burden among caregivers of children with chronic kidney disease in Assiut, Egypt
Source: J Egypt Public Health Assoc. 2020 Oct 7;95:27. doi: 10.1186/s42506-020-00058-7 (PMC7539246; doi:10.1186/s42506-020-00058-7)
Supplement: Supplementary file 1 — Additional file 1. [file 42506_2020_58_MOESM1_ESM.pdf]

كلية التربية
قسم أصول التربية

# مقياس المستوى الاجتماعي- الاقتصادي للأسرة
## (الصورة المعدلة)

إعداد
أ.د/ عبد التواب عبد اللاه عبد التواب
أستاذ أصول التربية
كلية التربية- جامعة أسيوط

| | |
|---|---|
| | سنة شهر يوم |
| **الاســـم:**........................... | **تاريخ اليوم:**............................... |
| **الوظيفة:**....................................... | **تاريخ الميلاد:**................................ |
| **سنة التخرج:**.................................. | **الســن:**............................. |
| **الإدارة التابع لها:** ......................... | **الجنــس: ذكر( ) أنثى ( )** |

**2010**

تهدف هذه الاستمارة إلى الحصول على بيانات شخصية لأغراض البحث العلمي، والرجاء الإجابة على البيانات التالية بكل صدق وأمانة وثق أن هذه البيانات سوف تحاط بالسرية التامة ولا يطلع عليها إلا الباحث.

**أولاً: بيانات عن الحالة التعليمية للأسرة:**

ضع علامة ( √) أمام المستوي التعليمي الذي بلغة كل من الأب والأم في المكان المناسب، مع ملاحظة أن سنوات الدراسة التي قضاها كل من الأب والأم ولم يحصل بها على شهادة لا تؤخذ في الاعتبار.

| | الأب | الأم |
|---|---|---|
| (1) لا يقرأ ولا يكتب. | ☐ | ☐ |
| (2) يقرأ ويكتب. | ☐ | ☐ |
| (3) حاصل على شهادة ابتدائية أو ما يعادلها. | ☐ | ☐ |
| (4) حاصل على شهادة إعدادية أو ما يعادلها. | ☐ | ☐ |
| (5) حاصل على شهادة ثانوية أو ما يعادلها. | ☐ | ☐ |
| (6) حاصل على شهادة جامعية أو ما يعادلها. | ☐ | ☐ |
| (7) حاصل على دراسات عليا (دبلوم أو ماجستير). | ☐ | ☐ |
| (8) حاصل على الدكتوراه. | ☐ | ☐ |

**ثانياً: بيانات عن الحالة المهنية للأسرة:**

أذكر مهنة كل من الأب والأم بالتفصيل؟

مهنة الأب:(.............................................................).

مهنة الأم:(.............................................................).

**ثالثاً: بيانات عن دخل الأسرة:**

ضع علامة (√) في المكان المناسب لدخل الأسرة في الشهر مع ملاحظة أن يشتمل الدخل على الدخل من الوظيفة بالإضافة إلى أي دخل من مصادر أخرى:

(1) أقل من 300 جنيا مصريا. ☐

(2) من 300 جنيها إلى أقل من 600جنيها. ☐

(3) من 600جنيه إلى أقل من 1000 جنيها. ☐

(4) من 1000 جنيها إلى أقل من 1800 جنيها. ☐

(5) من 1800 جنيها إلى اقل من 4000 جنيها. ☐

(6) أكثر من 4000 جنيها. ☐

**رابعاً: بيانات عن أسلوب الحياة التي تعيشها الأسرة:**

1–ضع علامة (√) أمام الإجابة التي تتفق مع مسكن الأسرة فيما يلي:

**أ– محل إقامة الأسرة:**

(1) في قرية ☐

(2) في مدينة ☐

**ب– نوع السكن:**

(1) إيجار ☐

(2) ملك

**جـ- الكثافة السكانية للأسرة(عدد الأفراد لكل غرفة):**

(1) ثلاثة أفراد فأكثر للغرفة الواحدة.

(2) فردان للغرفة الواحدة

(3) فرد للغرفة الواحدة

**2-ضع علامة (√) أمام العبارة التي تتفق مع أسرتك فيما يلي:**

- تشتري الأسرة الجريدة اليومية أو مجلة أسبوعية أو شهرية
- توجد بالمنزل مكتبة بها كتب( علمية – أدبية- ثقافية -دينية )
- تشترك الأسرة في نادي أو جمعية لممارسة بعض الأنشطة والهويات

**3- ضع علامة (√) أمام ما تمتلكه الأسرة من الآتي:**

- سيارة

(1) أجرة

(2) خاصة

- غسالة اتوماتيك
- غسالة أطباق
- ميكروويف
- جهاز كمبيوتر
- جهاز لاب توب
- دش لاستقبال القنوات التلفزيونية والفضائية
- تكييف
- كاميرا ديجتال

ملحق رقم (9)
مفتاح تصحيح مقياس المستوى الاجتماعى الاقتصادى للأسرة

مقياس المستوى الاجتماعى الاقتصادى للأسرة

يحتوى هذا المقياس على أربعة متغيرات أساسية، وكل متغير يتضمن مجموعة من المستويات أو البنود الفرعية، وقد وضعت المستويات على مقياس نقط متدرج يبدأ بالرقم (1) وينتهي برقم يمثل عدد المستويات. يعطي لكل مستوى درجة أو نقطة حسب ترتيبه في المقياس المتدرج، ثم تضرب هذه الدرجة في الوزن النسبي للمتغير الأساسي الذي يتضمن هذا المستوى، والدرجة الناتجة تعبر عن مستوى الأسرة في هذا المتغير.

**مع ملاحظة الآتي:**

1- في المتغيرات التى تتضمن بيانات عن مستوى الأب والأم مثل (التعلم والمهنة) تجمع درجة الأب ودرجة الأم الحاصل عليها كل منهما في المستويات الفرعية للمتغير، ثم تضرب هذه الدرجة في الوزن النسبي له.

2- الرقم الموجود بين القوسين – ناحية اليمين – يدل على ترتيب المستويات الفرعية لكل متغير على المقياس المتدرج، فالرقم (1) يدل على المستوى الأول، والرقم (2) يدل على المستوى الثاني... وهكذا. ويمثل هذا الرقم الدرجة التي يحصل عليها الفرد في المستوى الفرعي للمتغير الأساسي.

**وفيما يلي تقدير المتغيرات الأساسية والدرجات التي تحصل عليها:**

**أولاً: تقدير المستوى التعليمي**

يقسم المستوى التعليمي إلى ثمانية مستويات فرعية، وضعت على مقياس نقط يتدرج من (1 إلى 8)، ويعطي للمستوى الأول درجة أو نقطة، والمستوى الثاني درجتان أو نقطتان... وهكذا، ويعطي للفرد الدرجة المقابلة لمستوى التعليم الذي يحدده (✓) وفيما يلي المستويات الفرعية والدرجة التي يحصل عليها كل مستوى:

| المستويات | الدرجة |
|---|---|
| 1- لا يقرأ ولا يكتب | 1 |
| 2- يقرأ ويكتب | 2 |
| 3- حاصل على شهادة ابتدائية أو ما يعادلها | 3 |
| 4- حاصل على شهادة إعدادية أو ما يعادلها | 4 |
| 5- حاصل على شهادة ثانوية أو ما يعادلها | 5 |
| 6- حاصل على دراسات عليا (دبلوم أو ماجستير) | 6 |
| 7- حاصل على درجة الدكتوراه | 7 |

**ثانياً: تقدير المستوى المهني**

تصنف المهن إلى تسعة مستويات فرعية، وضعت على مقياس متدرج (من 1 إلى 9) يعطي المستوى الأول درجة، المستوى الثاني درجتان... وهكذا ويعطي للفرد الدرجة المقابلة للمستوى المهني الذي يحدده بذكره المهنة بالتفصيل.

**وفيما يلي مستويات المهن والدرجة التي يحصل عليها كل مستوى:**

**المستوى الأول: يتضمن المهن التالية:**

المهن غير الفنية وما في مستواها من العمال غير الفنيين وغير المهرة، والعاملين وغير العاملين بالحكومة والقطاع الخاص مثل: الباعة المتجولين، والخفراء (نظامي – خصوصي)، والعاملين بالنظافة (البوابين – عمال تشغيل المصاعد – وعمال الشحن، والحلاقين، والحانوتية، وما سحي الأحذية، والمكوجية، والطباخين،

والجرسونات، والعمل الزراعيين، وصيادي السمك، والمراكبية، والعاملين بمحطات البنزين، ونجاري المسلح، وملاحظي الجراج، والعاملين بالشحن (العتالين) والعاملين بالبلدية، والخدم في المنازل، وعمال التليفونات وعمال البناء.

**وتأخذ المهنة التي تقع في إطار هذا المستوى "درجة واحدة"**

**المستوى الثاني: ويتضمن المهن التالية:**

أصحاب المهن الحرفية البسيطة مثل: (الجزارين، والتجارين وصانعي الأحذية، وصغار تجار البقالة، والخردواتية، وتجار الدواجن، والبيض والغلال)، وأصحاب المهن الزراعية مثل: العامل الزراعي الذي يزرع أرضه، والعاملين بمرافق الخدمات مثل (رجال المرور ومساعدة الشرطة، ورجال الإسعاف)، وسائقي السيارات، وأصحاب المقاهي، والقومسنجية، وتجار الشنطة، وأصحاب الأعمال الكتابية مثل: (كتبة السجلات، وأمناء المخازن، والقبانية)، ومحصلي وسائل النقل مثل الكمسارية.

وتأخذ المهنة التي تقع في إطار هذا المستوى "درجتان"

**المستوى الثالث: ويتضمن المهن التالية:**

العمال المهرة وأصحاب المهن الفنية والمتوسطة مثل (الكهربائيين، ونجاري الموبيليا، وميكانيكية السيارات، والسباكين، والنقاشين، والمبيضين، والفكهانية، ومتعهدي الصحف، وأصحاب محالات الفراشة، والسماسرة، وتجار الورق، وتجار الأقمشة، والترزية، وأصحاب محلات البويات), وأصحاب الأعمال الذين يعملون فيها ويديرونها مثل (أصحاب المطاعم، والكافتيريا، والمخابز).

**وتأخذ المهنة التي تقع في إطار هذا المستوى "ثلاث درجات"**

**المستوى الرابع: ويتضمن المهن التالية:**

المهن الفنية المتوسطة والعاملين بالوظائف الكتابية والذين يحملون مؤهلا متوسطاً مثل: (محصلي المال، والصرافين، وماكسي الدفاتر ومراجعي الحسابات وأصحاب الوظائف الكتابية بالاعدادية، ومدرسي المرحلة الابتدائية، ووكلاء البريد، والمأذون الشرعي ومفتشي النقل والمواصلات، وسكرتارية المحاكم وأصحاب بعض الوظائف الفنية مثل: (فني الأشعة، والحكيمات بالمستشفيات والممرضات، وأخصائي النظارات، ومساعدي المهندسين) والمساعدين بالجيش ومن في مستواهم، وبعض أصحاب المهن الفنية فوق المتوسطة مثل (رؤساء مكاتب البريد، وأمناء السجل المدني، والسكرتارية ورؤساء القطارات) وكبار التجار مثل (أصحاب مصانع الطوب، ومحلات الإكسسوار، والجواهرجية).

**وتأخذ المهنة التي تقع في إطار هذا المستوى "أربع درجات"**

**المستوى الخامس:** ويتضمن المهن التالية:

العاملين بالدولة والذين يحملون مؤهلا فوق المتوسط: (مدرسي المرحلة الاعدادية، والذين يحملون مؤهلاً متوسطاً أو فوق المتوسط، ونظار التعليم الابتدائي، وموجهي التعليم الابتدائي، ومساعدي المهندسين، ورؤساء الأقسام بهيئة السكة الحديد، والفنانين التشكيليون، ومرشدي السفن التجارية). والموظفين التنفيذيين ومن في مستواهم مثل: (عمدة بلد، شيخ بلد)، والمرشدين السياحيين، وموظفي الجمارك، وموظفي الجمارك، وموظفى المطارات ورجال الدين (أئمة المساجد، ومفتشى الوعظ).

**وتأخذ المهنة التى تقع فى إطار هذا المستوى "خمس درجات"**

**المستوى السادس: ويتضمن المهن التالية:**

الموظفين بالحكومة والقطاع العام والخاص والحاصلين على مؤهل جامعي في مختلف التخصصات مثل المهندسين والأطباء، والصيادلة، والجيولوجيين والبيطريين، والمحاميين، ورؤساء الشئون القانونية) وصغار ضباط الشرطة والجيش (ملازم، ملازم أول، نقيب) ومأموري الضرائب، والمفتشين الماليين، والإداريين، ومديري الفنادق، ومدرسي التعليم الاعدادي والثانوي، وأصحاب المؤهلات العليا، وأخصائيون الكمبيوتر والحاسب الآلي ومهندسو الاتصالات السلكية واللاسلكية والإلكترونيات، ووكلاء المدارس الثانوية، والفنانين مثل: (الممثلين، الملحنين، ومخرجي السينما)، والصحفيين، ومذيعي وكالات الأنباء، والمعلقين في الصحف والإذاعات، والمضيفات في شركات الطيران، ومفتيشي التحقيقات ورؤساء الحسابات، ومفتشي المحاكم، والصحة والنيابة.

**وتأخذ المهنة التي تقع في إطار هذا المستوى "ست درجات"**

**المستوى السابع: ويتضمن المهن التالية:**

نظار التعليم الاعدادي والتعليم الثانوي، وموجهي المرحلة الثانوية، ومحامي مجلس الدولة، ورؤساء مجالس المدن، ومديري البنوك ومديري الجمارك، والمطارات وكبار الضباط (رائد – مقدم – عقيد)، ورجال القانون مثل: (القضاء، ورؤساء ووكلاء النيابة)، والمستشارين الطبيين بالقوات المسلحة، ورؤساء مكاتب حركات النقل، والمراقبين العاملين، ومفتشي الداخلية، ومديري العلاقات العامة، والباحثين مثل: (الباحثين في المراكز والهيئات الحكومية، والمعيدين والمدرسين المساعدين) ووكلاء المديريات.

**وتأخذ المهنة التي تقع في إطار هذا المستوى "سبع درجات"**

**المستوى الثامن: ويضمن المهن التالية:**

أصحاب المهن العلمية المتخصصة الحاصلين على درجة الدكتوراه مثل: أعضاء هيئة التدريس بالجامعات (مدرسين – أساتذة مساعدين) والمستشارين والخبراء في مختلف التخصصات، ومستشاري السفارات، والوزراء المفروضين بوزارة الخارجية، وأمناء الجامعات، ومديري المصانع الكبرى، وكبار ضباط الشرطة والجيش (عميد – لواء).

**وتأخذ المهنة التي تقع في إطار هذا المستوى "ثماني درجات"**

**المستوى التاسع: ويتضمن المهن التالية:**

أساتذة الجامعات، ورؤساء الأقسام، وعمداء الكليات، ونواب رؤساء الجامعات ورؤساء الجامعات، ووكلاء الوزراء، والوزراء، والسفراء والمحافظين.

**وتأخذ المهنة التي تقع في إطار هذا المستوى "تسع درجات"**

**ثالثاً: تقدير مستوى الدخل:**

يقسم الدخل إلى ستة مستويات فرعية، وضعت على مقياس نقط يتدرج (من 1 إلى 6) ويعطي المستوى الأول درجة واحدة والمستوى الثاني درجتان ...وهكذا. ويعطي للفرد الدرجة المقابلة لمستوى الدخل الذي يحدده بعلامة (✓).

**وفيما يلي مستويات الدخل والدرجة التي يحصل عليها كل مستوى:**

| المستويات | الدرجة |
|---|---|
| 1- أقل من 300 جنيهاً مصرياً | 1 |
| 2- من 300 جنيه إلى أقل من 600 جنيه | 2 |
| 3- من 600 جنيه إلى أقل من 1000 جنيه | 3 |

4- من 1000 جنيه إلى أقل من 1800 جنيه 4
5- من 1800 جنيه إلى أقل من 4000 5
6- أكثر من 4000 جنيه 6

**رابعاً: تقدير مستوى أسلوب الحياة:**

يحتوى هذا البعد على ثلاثة متغيرات فرعية، وكل متغير يحتوى على مجموعة من البنود، ويتم تقدير مستوى أسلوب الحياة كألاتي:

**1- مستوى السكن:**

**أ- محل الإقامة:** تعطي درجة واحدة للمقيم في قرية، ودرجتان للمقيم في مدينة.

**ب- نوع السكن:** تعطي درجة واحدة إذا كان المسكن إيجار وتعطي درجتان إذا كان المسكن ملك.

**جـ- الكثافة السكانية:** تعطى درجة واحدة إذا كانت الكثافة السكانية للأسرة ثلاثة أفراد أو أكثر للغرفة الواحدة، ودرجتان إذا كانت فردين للغرفة الواحدة، وثلاثة درجات إذا كانت فرداً واحداً للغرفة الواحدة.

**2- المستوى الثقافي:**

تعطى درجة واحدة لكل بند من بنود المستوى الثقافي.

**3- ممتلكات الأسرة الخاصة:**

تعطي درجة واحدة إذا كانت الأسرة تمتلك سيارة لاستخدامها تاكسي أجرة ودرجتان إذا كان تمتلكها للاستخدام الخاص، وتعطي البنود الباقية درجة واحدة فقط.

**طريقة حساب الدرجة الكلية للمستوى الاجتماعي – الاقتصادي:**

1- تجمع الناتجة في الوزن النسبي له (7.33) لتعبر عن المستوى التعليمي درجة المستوى التعليمي للأب والأم، ثم تضر الدرجة للأسرة.

2- تجمع درجة المستوى المهني للأب والأم، ثم تضرب الدرجة الناتجة في الوزن النسبي له (6.91) لتعبر عن المستوى المهني للأسرة.

3- تضرب درجة مستوى دخل الأسرة في الوزن النسبي له (4.86) لتعبر عن مستوى دخل الأسرة.

4- تجمع درجات (مستوى السكن، والمستوى الثقافي، والممتلكات الخاصة) ثم تضرب هذه الدرجة في الوزن النسبي لمتغير أسلوب الحياة (3.12) لتعبر عن مستوى أسلوب الحياة التى تعيشها الأسرة.

5- تستخدم المعادلة التالية للحصول على المستوى الاجتماعي – الاقتصادي مقدراً بالدرجات:

**حيث:**

$$س = 7.33 س_1 + 6.91 س_3 + 3.12 س_4$$

**س = المستوى الاجتماعي – الاقتصادي للاسرة.**

**$س_1$ = الدرجة الحاصل عليها الفرد في المستويات الفرعية لمتغير التعليم.**

**$س_2$ = الدرجة الحاصل عليها الفرد في المستويات الفرعية لمتغير المهنة.**

**$س_3$ = الدرجة الحاصل عليها الفرد في المستويات الفرعية لمتغير الدخل.**

**$س_4$ = الدرجة الحاصل عليها الفرد في المستويات الفرعية لمتغير أسلوب الحياة.**

6- ويمكن استخدام هذه الدرجة في الحالتين التاليتين:

**أولاً: عند استخدام المستوى الاجتماعي – الاقتصادي كعامل تثبيت:**

في هذه الحالة يطبق المقياس على العينة المراد دراستها، ويصحح المقياس والدرجة الحاصل عليها الفرد في المقياس تعبر عن مستواه الاجتماعي، الاقتصادي، ويمكن للباحث التعامل مع هذه الدرجة الخام عند تثبيت هذا المتغير.

**ثانياً: عند استخدام المستوى الاجتماعي – الاقتصادي كعامل متغير:**

**في هذه الحالة تتبع الخطوات التالية:**

أ- يطبق مقياس (S.E.S) على أفراد العينة، وتحسب الدرجة الكلية الحاصل عليها الفرد في المقياس.

ب- يحسب المتوسط الحاسبي، والانحراف المعياري لدرجات أفراد العينة في المقياس.

جـ- يتم تحديد المستوى الطبقي لأفراد العينة باستخدام منحنى التوزيع الاعتدالي حيث توزيع درجات أفراد العينة على منحنى التوزيع الاعتدالي، وتحدد المستويات الطبقية كالآتي:

**الطبعة الدنيا:** وتتمثل في الأفراد الحاصلين على درجات تقع في المدن الذي يقل عن المتوسط بمقدار (1- انحراف معياري).

**الطبقة المتوسطة:** وتتمثل في الأفراد الحاصلين على الدرجات التي تقع في المدن بين (المتوسط + انحراف معياري، والمتوسط – انحراف معياري).

**الطبقة العليا:** وتتمثل في الأفراد الحاصلين على درجات تقع في المدى الذي يزيد عن المتوسط بمقدار (1 + انحراف معياري).

والشكل التالي يوضح تحديد المستويات الطبقية باستخدام منحنى التوزيع الاعتدالي:

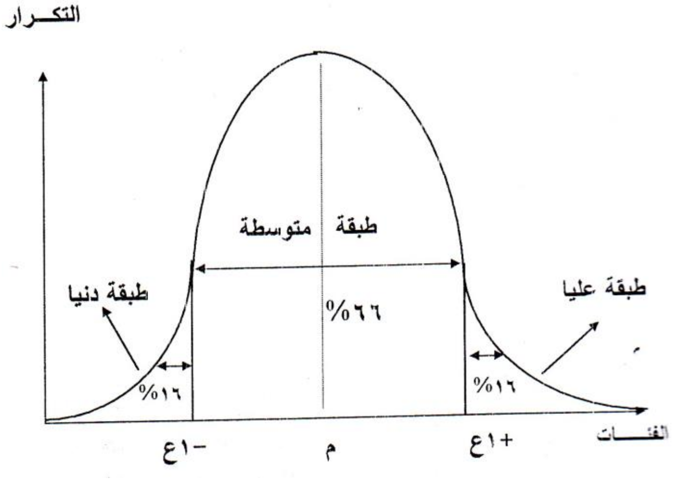

ويراعي عند استخدام هذه الطريقة استبعاد الدرجات التي تقع على الحد الفاصل بين مساحات المنحنى ضماناً للدقة والموضوعية في تحيد المستويات الطبقية.

7- وإذا كانت طبيعة الدراسة تحتاج إلى أكثر من ثلاثة مستويات طبقية مثل: (طبقة عليا – طبقة عليا دنيا...وهكذا) تستخدم أساليب إحصائية أخرى، فيمكن استخدام الخواص الاحصائية للتكرار المتجمع الصاعد، أو أية أساليب إحصائية أخرى في الحصول على مستويات تتفق مع طبيعة الدراسة.
