# Supplementary material for: Family impact and economic burden among caregivers of children with chronic kidney disease in Assiut, Egypt
Source: J Egypt Public Health Assoc. 2020 Oct 7;95:27. doi: 10.1186/s42506-020-00058-7 (PMC7539246; doi:10.1186/s42506-020-00058-7)
Supplement: Supplementary file 2 — Additional file 2. [file 42506_2020_58_MOESM2_ESM.pdf]

الرقم الشخصي:_______________________

التاريخ:_____________________________

PedsQL ™

استبيان فحص التأثير على العائلة

Version 2.0 - Arabic (Israel)

تقرير الوالدين

تعليمات

عائلات الأولاد تواجه أحيانا صعوبات خاصة بسبب صحّة أبنائهم. في الصفحة التالية قائمة بالأمور التي قد تكون مشكلة لك **أنت**. الرجاء أن تُخبرنا عن **حجم المشكلة** الناتجة **لك** عن كل واحدة منها خلال **الأيام السبعة الماضية** بوضع دائرة حول:

صفر إذا هي ليست مشكلة **أبداً**
1 إذا هي **ليست مشكلة في معظم الأحيان**
2 إذا هي مشكلة في **بعض الأحيان**
3 إذا هي مشكلة **في أحيان كثيرة**
4 إذا هي **مشكلة في معظم الأحيان**

لا توجد إجابات صحيحة أو خاطئة.
إذا لم تفهم السؤال، الرجاء طلب المساعدة.

J. W. Varni, Ph.D. 1998 © حقوق الطبع
جميع الحقوق محفوظة
غير مصرّح بإعادة الطبع بدون إذن

في *الأيام السبعة* الماضية، ما حجم المشكلة التي واجهتها *أنت* نتيجة صحّة ابنك؟

**الصحة الجسمانية** *(مشاكل بخصوص ...)*	ليست مشكلة أبدا	ليست مشكلة في معظم الأحيان	هي مشكلة في بعض الأحيان	هي مشكلة في أحيان كثيرة	هي مشكلة في معظم الأحيان
1. أشعر بالتعب خلال اليوم	0	1	2	3	4
2. أشعر بالتعب حين أستيقظ في الصباح	0	1	2	3	4
3. أشعر بالتعب الشديد ولا أقدر على فعل أشياء أرغب في فعلها	0	1	2	3	4
4. تكون لدي أوجاع رأس	0	1	2	3	4
5. أشعر بالضعف الجسدي	0	1	2	3	4
6. أشعر بأن معدتي متقلبة	0	1	2	3	4

**الحالة المعنوية** *(مشاكل بخصوص ...)*	ليست مشكلة أبدا	ليست مشكلة في معظم الأحيان	هي مشكلة في بعض الأحيان	هي مشكلة في أحيان كثيرة	هي مشكلة في معظم الأحيان
1. أشعر بالهمّ	0	1	2	3	4
2. أشعر بالحزن	0	1	2	3	4
3. الشعور بالغضب	0	1	2	3	4
4. أشعر بالإحباط	0	1	2	3	4
5. أشعر أنني عاجز أو فاقد للأمل	0	1	2	3	4

**الأنشطة الاجتماعية** *(مشاكل بخصوص ...)*	ليست مشكلة أبدا	ليست مشكلة في معظم الأحيان	هي مشكلة في بعض الأحيان	هي مشكلة في أحيان كثيرة	هي مشكلة في معظم الأحيان
1. أشعر أني وحيد وبعيد عن الآخرين	0	1	2	3	4
2. أجد مشكلة في الحصول على الدعم من الآخرين	0	1	2	3	4
3. من الصعب إيجاد الوقت للنشاطات الاجتماعية	0	1	2	3	4
4. لا طاقة كافية لدي للقيام بالنشاطات الاجتماعية	0	1	2	3	4

**إرهاق ذهني (تعب بال)** *(مشاكل بخصوص ...)*	ليست مشكلة أبدا	ليست مشكلة في معظم الأحيان	هي مشكلة في بعض الأحيان	هي مشكلة في أحيان كثيرة	هي مشكلة في معظم الأحيان
1. من الصعب عليّ إبقاء تركيزي في الأشياء	0	1	2	3	4
2. من الصعب عليّ تذكّر ما يخبرني به الناس	0	1	2	3	4
3. من الصعب عليّ تذكّر ما سمعته قبل لحظات قليلة	0	1	2	3	4
4. من الصعب عليّ التفكير بسرعة	0	1	2	3	4
5. أجد مشكلة في تذكّر ما كنت أفكّر فيه قبل لحظات قليلة	0	1	2	3	4

**التواصل** *(مشاكل بخصوص ...)*	ليست مشكلة أبدا	ليست مشكلة في معظم الأحيان	هي مشكلة في بعض الأحيان	هي مشكلة في أحيان كثيرة	هي مشكلة في معظم الأحيان
1. أشعر أن الآخرين لا يفهمون وضع عائلتي	0	1	2	3	4
2. من الصعب عليّ أن أتحدّث مع الآخرين عن صحّة ابني\ابنتي	0	1	2	3	4
3. من الصعب عليّ أن أقول للأطباء والممرضين ما أشعر به	0	1	2	3	4

J. W. Varni, Ph.D. 1998 © حقوق الطبع
جميع الحقوق محفوظة
غير مصرّح بإعادة الطبع بدون إذن

*في **الأيام السبعة** الماضية، ما حجم المشكلة التي واجهتها **أنت** نتيجة صحّة ابنك؟*

**القلق** *(مشاكل بخصوص ...)*	ليست مشكلة أبدا	ليست مشكلة في معظم الأحيان	هي مشكلة في بعض الأحيان	هي مشكلة في أحيان كثيرة	هي مشكلة في معظم الأحيان
1. أقلق حول ما إذا كانت العلاجات الطبية التي يأخذها ابني\ابنتي ناجحة أم لا	0	1	2	3	4
2. أقلق من الأعراض الجانبية التي تسببها الأدوية \ العلاجات الذي يأخذها ابني\ابنتي	0	1	2	3	4
3. أقلق من كيف ستكون ردّة فعل الآخرين مع وضع ابني\ابنتي	0	1	2	3	4
4. أقلق من كيفية تأثير مرض ابني\ابنتي على باقي أفراد عائلتي	0	1	2	3	4
5. أقلق من مستقبل ابني\ابنتي	0	1	2	3	4

تعليمات

في الصفحة التالية قائمة بالأمور التي قد تكون مشكلة **لعائلتك.** الرجاء أن تُخبرنا عن **حجم المشكلة** الناتجة **لعائلتك** عن كل واحدة منها خلال **الأيام السبعة** *الماضية*.

*في **الأيام السبعة** الماضية، ما حجم المشكلة التي واجهتها **عائلتك** نتيجة صحّة ابنك؟*

**الأنشطة اليومية** *(مشاكل بخصوص ...)*	ليست مشكلة أبدا	ليست مشكلة في معظم الأحيان	هي مشكلة في بعض الأحيان	هي مشكلة في أحيان كثيرة	هي مشكلة في معظم الأحيان
1. النشاطات العائلية تأخذ وقتاً وجهداً أكبر	0	1	2	3	4
2. هناك صعوبة في إيجاد الوقت اللازم لإنهاء المهام المنزلية	0	1	2	3	4
3. شعور بالتعب الشديد لدرجة تمنع العائلة من إنهاء المهام المنزلية	0	1	2	3	4

**العلاقات العائلية** *(مشاكل بخصوص ...)*	ليست مشكلة أبدا	ليست مشكلة في معظم الأحيان	هي مشكلة في بعض الأحيان	هي مشكلة في أحيان كثيرة	هي مشكلة في معظم الأحيان
1. هناك نقص في التواصل بين أفراد العائلة	0	1	2	3	4
2. هناك صراع بين أفراد العائلة	0	1	2	3	4
3. هناك صعوبة في اتخاذ القرارات كعائلة بصورة جماعية	0	1	2	3	4
4. هناك صعوبة في حل مشاكل العائلة بصورة جماعية	0	1	2	3	4
5. هناك توتّر بين أفراد العائلة	0	1	2	3	4

J. W. Varni, Ph.D. 1998 © حقوق الطبع
جميع الحقوق محفوظة
غير مصرّح بإعادة الطبع بدون إذن
