# Supplementary material for: Family impact and economic burden among caregivers of children with chronic kidney disease in Assiut, Egypt
Source: J Egypt Public Health Assoc. 2020 Oct 7;95:27. doi: 10.1186/s42506-020-00058-7 (PMC7539246; doi:10.1186/s42506-020-00058-7)
Supplement: Supplementary file 3 — Additional file 3. [file 42506_2020_58_MOESM3_ESM.pdf]

PedsQL TM 2.0 Family Impact Module

The Parent report of the **PedsQL**TM 2.0 Family impact Module is composed of 36 items comprising
8 dimensions.

DESCRIPTION OF THE FAMILY IMPACT MODULE:

**Dimensions**	**Number of Items**	**Cluster of Items**	**Reversed Scoring**	**Direction of Dimensions**
Physical Functioning	6	1-6	1-6	Higher scores indicate better functioning.
Emotional Functioning	5	1-5	1-5	
Social Functioning	4	1-4	1-4	
Cognitive Functioning	5	1-5	1-5	
Communication	3	1-3	1-3	
Worry	5	1-5	1-5	
Daily Activities	3	1-3	1-3	
Family Relationships	5	1-5	1-5	

SCORING OF DIMENSIONS:

**Item Scaling**	5-point Likert scale from 0 (Never) to 4 (Almost always)
**Weighting of Items**	No
**Extension of the Scoring Scale**	Scores are transformed to a 0 to 100 scale.
**Scoring Procedure**	**Step 1: Transform Score**<br>Items are reversed scored and linearly transformed to a 0-100 scale as follows: 0=100, 1=75, 2=50, 3=25, 4=0<br>**Step 2: Calculate Scores by Dimensions**<br>• If more than 50% of the items in the scale are missing, the scale scores should not be computed,<br>• Mean score = Sum of the items over the number of items answered.<br>**Step 3: Total Scores**<br>• The Total Score is the sum of all 36 items divided by the number of items answered<br>• The Parent HRQL Summary Score (20 items) is computed as the sum of the items divided by the

	number of items answered in the Physical, Emotional, Social, and Cognitive Functioning scales.<br>• The Family Functioning Summary Score (8 items) is computed as the sum of the items divided by the number of items answered in the Daily Activities and family Relationships scales.

PedsQL TM 2.0 Family Impact Module

**Interpretation and Analysis of Missing Data**	If more than 50% of the items in the scale are missing, the Scale Scores should not be computed.<br>If 50% or more items are completed: Impute the mean of the completed items in a scale.
